# Patient experiences of remote consulting with chronic fatigue syndrome/myalgic encephalomyelitis and fibromyalgia: a qualitative study

**DOI:** 10.3399/BJGPO.2024.0079

**Published:** 2025-02-26

**Authors:** Helen Leach, Abi Eccles, Carolyn A Chew-Graham, Helen Atherton

**Affiliations:** 1 Unit of Academic Primary Care, University of Warwick, Coventry, UK; 2 School of Medicine, Keele University, Keele, UK; 3 Primary Care Research Centre, University of Southampton, Southampton, UK

**Keywords:** remote consulting, fatigue syndrome, chronic, fibromyalgia, primary health care

## Abstract

**Background:**

Remote and digital consulting in primary care has rapidly expanded since March 2020. It is important to understand patient experiences, particularly for those living with complex long-term conditions, to identify how care can best be delivered, including within the remote space.

**Aim:**

To explore the experiences of people living with chronic fatigue syndrome/myalgic encephalomyelitis (CFS/ME) and fibromyalgia when consulting remotely in primary care.

**Design & setting:**

Semi-structured interviews with patients living with CFS/ME and fibromyalgia in general practice in England.

**Method:**

Semi-structured interviews were carried out with 13 participants. The interviews were transcribed and analysed thematically according to a Foucauldian theoretical framework.

**Results:**

All participants highlighted needing to feel believed by clinicians. Many reported difficulties with telephone and online consulting owing to the lack of physical communication. Positive outcomes were reported when there was a good relationship with a clinician. Continuity in care and recognising the complexity of these conditions were also considered important.

**Conclusion:**

This study allowed people living with CFS/ME and fibromyalgia to describe their experiences when consulting remotely. Participants highlighted needing to feel listened to and felt they benefited from an ongoing relationship with a clinician although this was difficult to achieve when consulting remotely. Some advantages of remote consulting were reported, particularly when symptoms were troublesome. Flexible access systems, with a range of consultation modalities or preferred clinician(s) availability, could improve healthcare encounters, particularly given the increased use of remote consulting in primary care.

## How this fits in

Previous research has explored how remote consulting is experienced by varying patient groups. This study explores how it is experienced by people living with chronic fatigue syndrome/myalgic encephalomyelitis (CFS/ME) and fibromyalgia, which are complex, long-term conditions. Importance was placed on being listened to, building relationships with clinicians, and continuity of care. Clinicians should prioritise these aspects to enhance patient care.

## Introduction

Primary care has seen a rise in the use of alternatives to face-to-face consulting, including synchronous (telephone and video) and asynchronous (email, text, or online portal) modalities. These alternatives, promoted in part by national policy, sought to support general practice and moved with wider societal changes integrating digital technology into services. While the introduction had been steady and a minor proportion of the workload,^1^ the COVID-19 pandemic resulted in a sudden shift to a predominantly remote consulting model in March 2020. The number of face-to-face consultations has subsequently increased but remote modalities will likely remain prominent going forward.^2^

Literature has considered the experiences and effects of remote consulting for healthcare needs. For many patients, these modalities are acceptable, effective, and enhance convenience.^3,4^ However, there are concerns remote consulting may be less suited for delivering personalised or patient-centred care.^5,6^ Reasons suggested for this have included fundamental differences in the ways patients and clinicians interact in the digital or remote space.^5^

Chronic fatigue syndrome/myalgic encephalomyelitis (CFS/ME) and fibromyalgia are complex, long-term conditions. The symptomologies are broad, not limited to chronic pain and fatigue, and impact on quality of life.^7,8^ Currently, there is no clear aetiology nor treatment for either condition, further contributing to difficulties and challenges. Current recommendations highlight the importance of utilising personalised care to support function and recovery, and building a therapeutic alliance with patients.^9,10^ While patient-centred care should be a quintessential element of all primary care consultations, building a relationship, recognition of the individual experience, and shared decision making are crucial given the uncertainties and challenges of these conditions.

Much has been written on the experiences of people with fibromyalgia or CFS/ME and the struggles for their illness experience, and diagnosis, to be legitimised by the medical profession.^11–13^ This has often been linked to the lack of defined pathophysiological mechanisms, inconsistent diagnostic criteria, and the dynamic between biopsychosocial and biomedical models of understanding. It is therefore important to consider the roles of, and interactions between, ‘clinician’ and ‘patient’ within the consultation, along with consideration of how previous experiences and factors contribute to future encounters.

Little is currently known about the experiences of remote consulting for people living with CFS/ME and fibromyalgia in primary care. Research has examined remote consulting in generalised chronic pain and there are also evaluations of delivering online therapies.^14,15^ Remote consulting has been considered beneficial in the context of substantial fatigue and post-exertional malaise.^16^ However, little exploration has focused on the experience of the person within the remote consultation and the impact on patient-centred care. Given the well-documented challenges, examining the impact of remote consultations is crucial for supporting people with these complex conditions.

## Method

This was a qualitative study conducted with patients about care in general practice settings. A theoretical framework was applied to aid interpretation of the findings. All participants provided written or verbal consent after information documentation was shared, the study was explained, and any questions answered.

### Patient and public involvement (PPI)

Multiple workshops included the voices of people living with CFS/ME and fibromyalgia in the development process. An initial workshop was held with four participants to discuss the study, recruitment, and areas to explore within the interviews based on lived experience. A subsequent workshop with three participants reviewed and iteratively modified the topic guide. Discussion also covered how to conduct interviews with this patient population to account for symptoms such as brain fog, fatigue, and post-exertional malaise. A final workshop with two participants reviewed transcript extracts with interpretations and findings integrated into the coding and wider thematic analysis.

### Recruitment and sampling

Recruitment was conducted with support from the Clinical Research Network (CRN) West Midlands. This region demonstrated a mix of population demographics, including rurality, a range of practice sizes, and deprivation scores. Potential participants were screened by their general practices and contacted by post by the practice with a participant information sheet and pre-paid reply envelope addressed to the research team. After interested participants contacted the research team via post, further screening was conducted by HL via telephone (a GP trainee and Academic Clinical Fellow) before inclusion.

Participants were required to be aged >18 years, have a diagnosis of CFS/ME and/or fibromyalgia, and have had at least one remote consultation with a primary care clinician in the past 3 months related to their CFS/ME or fibromyalgia. Given the complexities of these diagnoses, it was recognised that this final inclusion criteria may be difficult to ascertain from notes alone. This two-step screening was taken to ensure participants would be able to speak about consulting remotely about their condition.

Potential participants were purposively selected to include a range of ages, sexes, socioeconomic status, and diagnoses, that is, CFS/ME and/or fibromyalgia (Table 1). Despite extending the recruitment period and approaching practices with diverse patient demographics, only one Black British Caribbean participant expressed interest.

**Table 1.** Demographics of participants

Participant number	Diagnosis	Age group	Sex	Ethnicity	Practice deprivation decile (IMD data)
P1	Fibromyalgia	60–69	F	White British	Fifth more deprived
P2	Fibromyalgia	40–49	F	White British	Least deprived
P3	Fibromyalgia and CFS/ME	70–79	M	White British	Fifth more deprived
P4	CFS/ME	50–59	F	White British	Third less deprived
P6	Fibromyalgia	40–49	F	White British	Fifth more deprived
P7	CFS/ME	40–49	M	White British	Fifth more deprived
P8	Fibromyalgia	40–49	M	White British	Fifth more deprived
P9	Fibromyalgia	60–69	F	White British	Fifth more deprived
P10	CFS/ME	30–39	F	White British	Fifth more deprived
P12	Fibromyalgia	70–79	F	White British	Third less deprived
P13	Fibromyalgia and CFS/ME	40–49	F	White British	Third less deprived
P14	Fibromyalgia and CFS/ME	50–59	F	White British	Third less deprived
P15	Fibromyalgia and CFS/ME	50-–59	F	Black British Caribbean	Most deprived

CFS/ME = chronic fatigue syndrome/myalgic encephalomyelitis, IMD = Index of Multiple Deprivation.

### Data generation

HL conducted semi-structured interviews with participants between January and July 2023. Interviews were conducted via phone, video, or face to face based on participant preference. Flexibility was provided to account for CFS/ME and fibromyalgia symptomology, including rescheduling, pausing, or terminating early. All interviews were audio-recorded.

Participants were asked about their experiences of consulting remotely with primary care clinicians. Experiences consulting face to face or with secondary care specialists were also explored as comparators. The topic guide was developed to be broad and it was semi-structured to give participants scope to speak more generally about their experiences. It was modified iteratively during the interview process and with the PPI group.

### Data analysis and theoretical framework

The interviews were de-identified, then transcribed and imported into NVivo (version 10) for management. Data were analysed according to Braun and Clarke’s six-step process of thematic analysis.^17^ HL familiarised herself with all transcripts and developed initial codes. The codes and themes were developed during regular meetings with the wider research team, made up of experienced qualitative health researchers. The team was comprised of two primary healthcare researchers, one with particular expertise in remote consulting, and an academic clinician with research experience in CFS/ME.

Coding was undertaken deductively, underpinned by a Foucauldian theoretical framework. Foucauldian theory predominantly focuses on ‘power’ in its many guises and forms. Foucault wrote extensively on how power manifests itself within a medical context with their concepts of the ‘medical gaze’ and ‘power-knowledge’, acknowledging the link between knowledge and power.^18,19^ Foucault’s genealogical approach, or a ‘history of the present’, considers how previous experiences or factors influence those described.^19^ The concepts of ‘power’, building on previous work in remote consulting,^20^ and ‘genealogy’ shaped the coding approach and theme development.

## Results

**Figure 1.**
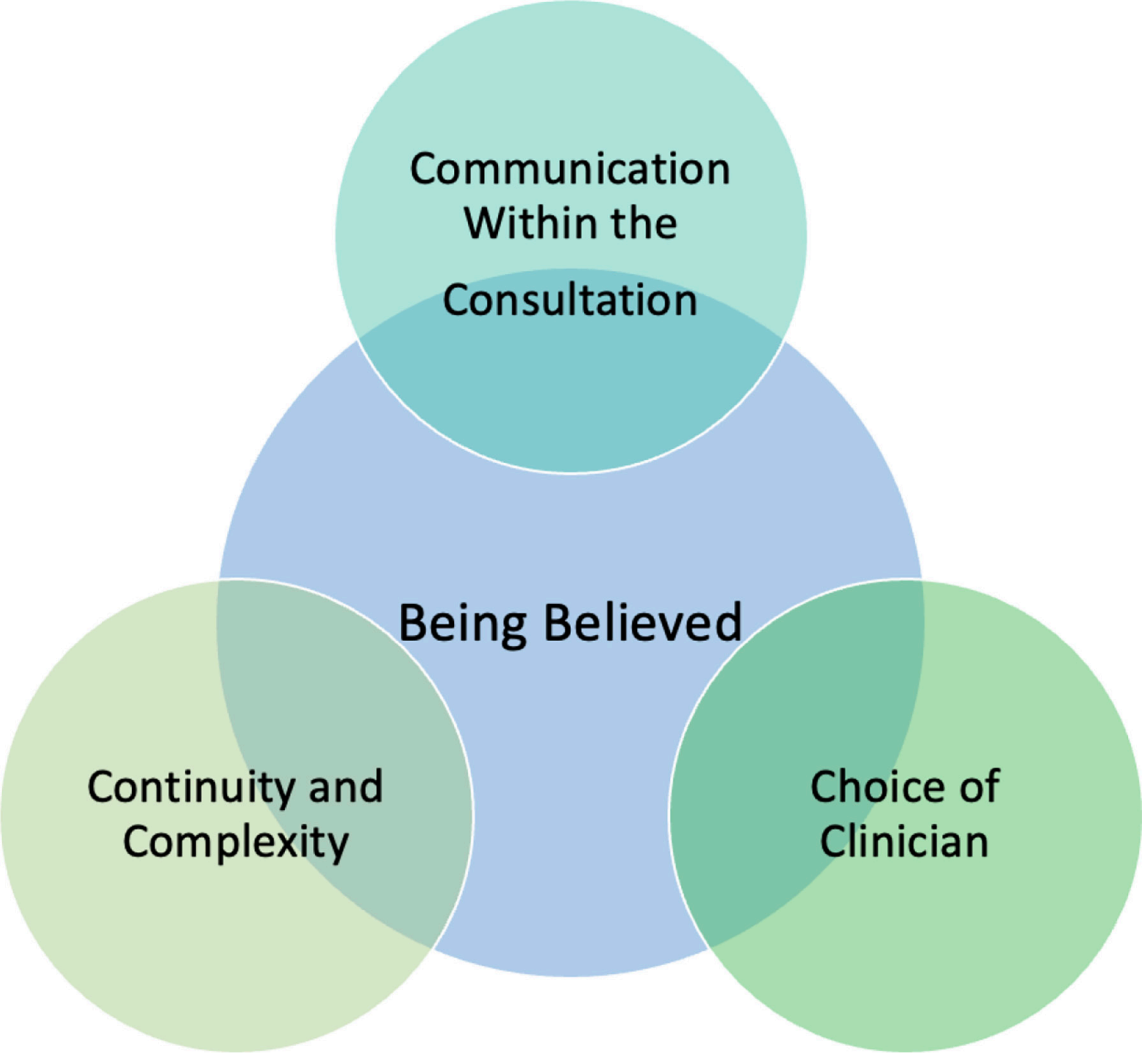
The themes that emerged from the semi-structured interviews

Thirteen participants were recruited (Table 1). An overarching theme of being believed when accessing health care was identified. The following three sub-themes were developed: communication within the consultation; choice of clinician; and continuity and complexity (Figure 1). Data extracts are included to illustrate the themes and they are denoted by participant number.

### Being believed

Throughout the interviews, participants shared a want to feel believed in their illness experience. Many spoke extensively of difficult experiences living with CFS/ME and fibromyalgia. Some expressed a perspective that clinicians did not believe in these conditions, compounded by their sometimes-hidden nature or lack of understanding, and highlighted an imbalance of ‘power-knowledge’:

*'Pain and some stuff is hard if you can’t see it and some people don’t see it do they, do you know what I mean? You just wanna say, “Get in my body and feel it and then you’ll know what I mean.”*' (P1)

Many participants explained they would independently self-manage their condition, only contacting practices when there was a specific medical or administrative need but sometimes reached ‘crisis point’ before seeking care. This was often underpinned by not wanting to *‘waste*’ their own or the clinicians’ time, particularly if they felt they risked a negative healthcare encounter (either not receiving the care they felt needed or a clinician unable to help):

*'… I don’t go to see them unless it’s absolutely necessary. I wanna make that absolutely clear … I only go and see the doctor when it’s absolutely necessary and I’m really, really in pain. Or it’s something that I think could be quite serious.'* (P3)

Viewing the sub-themes through the lens of validity shows how the participants in this study were impacted by their previous encounters with healthcare professionals. This genealogy was seen in how participants approached clinicians; many spoke of feeling disbelieved by health professionals, sometimes for many years preceding, with these experiences underpinning any future consulting:

*'It* [remote consulting] *works well as long as you’ve got the right support at the other end of the phone. As long as you’ve got the right team that understands, that don’t think, “Oh, they’ve been diagnosed with fibromyalgia, because they’ve given this diagnosis because, you know, we don’t know what else to call it.”'* (P6)

More positive encounters were reported where participants felt clinicians listened to and believed their symptoms and diagnosis, extending to the experiences of remote consulting, and choice of clinician.

### Communication within the consultation

Many participants shared positive and negative experiences when consulting remotely. Once access to an appointment had been gained, a remote consultation removed the need to attend the surgery and was regarded positively by some. However, telephone consultations were challenging and unpredictable without a set appointment time:

*'Obviously, travelling to doctor surgeries can be problematic … sometimes telephone calls can be very helpful, if you’re having a phone consultation, because it means you’re not having to go out.'* (P8)

All participants highlighted the importance of feeling listened to during their consultation and many shared experiences where this had not happened, although not limited to those undertaken remotely. There were mixed views as to how the illnesses and symptoms would be perceived across varying consultation modalities. Both video and face-to-face appointments were felt to reinforce the legitimacy of symptoms through ‘*showing*’ their symptoms and condition, highlighting the power held by a clinician within the consulting space:

*'… to actually look somebody in the face and let them see the pain in your face is much better than someone’s voice over the phone I feel.*' (P14)

Participants recognised physical examination as important, not only for a diagnosis but also for reassurance and contributing towards feeling listened to. Video consulting was seen to mitigate some of the concerns about non-verbal cues and allow some examination but was infrequently used:

*'… the doctor sent me a link and then I could send a video over to them … which I really liked, I thought that was wicked … because, because it was a physical thing that I wanted to show the doctor, you know, my twitching.*' (P7)

Relying on verbal communication within the remote space, particularly via telephone, was noted as difficult for those dealing with sensory disturbance or sensitivities. Use of loudspeaker, distracting keyboard noises, and bright screens detracted from the quality of the consultation:

*'… previous conversations with GPs that have been, where they are clearly on loudspeaker and they sound like 1 000 000 miles away and they're typing as well … But obviously it’s difficult when you’re sat opposite that GP, they tend to be just sort of typing away anyway. But sometimes when you … that’s all you can hear on the phone. It’s perhaps a little bit off-putting*.' (P10)

Physical presence, and the ability to communicate non-verbally or without distraction, were important but felt more challenging in a remote setting.

### Choice of clinician

Many participants spoke of specific clinicians during the interviews who listened to their experiences and appeared to understand the diagnoses of CFS/ME and fibromyalgia:

*'I had one doctor that was very good that actually helped me get the diagnosis of fibromyalgia and the chronic fatigue. And I built a relationship with him, and so I was able to ... I had telephone appointments with him as well as face to face.'* (P13)

A remote consultation, particularly online or via telephone, risked a participant not knowing who they were consulting with either ahead of time or during the encounter itself. For those who had had a constructive telephone encounter, this again was often dependent on whether they had felt listened to. This interweaved with the genealogy of face-to-face encounters; face to face was not innately better if still with a clinician who appeared not to listen or understand CFS/ME and fibromyalgia, with ‘power-knowledge’ remaining unbalanced:

*'Well, what if the doctor that I’m speaking to is one of those people that sees fibro as, as a dustbin diagnosis and doesn’t actually think there’s anything wrong with me? … And you can’t really tell that on the phone.*' (P2)

*'It doesn’t matter if I have to be in a room with someone or whether we do it over the phone, as long as we can have a conversation or two-way conversation. I’m OK with that.*' (P4)

The interface between access, remote consulting, and choice of clinician raised concerns. Many participants described urgent access as being via telephone or online by default and reported it being more difficult to choose a clinician to consult with. This risked consulting with an unknown clinician, with unknown understanding of CFS/ME or fibromyalgia, while highly symptomatic, without physicality to validate symptoms. These concerns were often again driven by previous experience and further highlighted feelings of powerlessness when these illnesses were perceived to be viewed negatively by clinicians:

*'And I, if I had gone through to the duty doctor, would the duty doctor have understood all the complexities, you know, like I said, everyone’s got their own, you know, story*.' (P8)

In contrast, one participant reported not having a preferred clinician. During the interview, she spoke of both feeling able to articulate herself and advocate within a consultation but also of not wanting to be seen as a ‘*hypochondriac*’ or a ‘*nuisance*’:

*'So I’m ok with seeing new people. Yeah, I’m ok with it if I feel that they’re taking what I say seriously or they’re helping me manage it. Because I always say to them, just need more information, I understand you’re not gonna cure me*.' (P15)

Positive experiences were contingent on being taken seriously or working in partnership together; while more frequently associated with preferred clinicians, this participant identified how it could still occur outside of an ongoing relationship but in the context of both medical and lived experience sharing ‘power-knowledge’ equally.

### Continuity and complexity

Consulting with the same clinician was perceived very positively by participants for being understood at an individual level. Relationships were felt to develop more easily when consulting face to face. Remote consultations were felt to benefit from a pre-existing relationship, although one participant reported an experience where a clinician being familiar with her notes was sufficient:

*'So it’s contingent on having that relationship with the GP. Or in place of that, the GP familiarising themselves with your notes, which is what this last GP did. He’d read through all of my notes and understood why I was calling. And so that’s why I felt the appointment went really well.*’ (P13)

Participants responded positively to clinicians who continued to build relationships by following up after a consultation or contacting them for a routine review. Telephone consultations were seen to have a role for this ‘*checking-in*’. Developing these relationships was seen to move both clinician and person with CFS/ME or fibromyalgia into more equal standing within the consultation; two participants spoke of ‘*working together*’ and ‘*being on a journey with*’ their clinicians. ‘Power-knowledge’ was not just limited to knowledge of CFS/ME or fibromyalgia, but also knowledge of that specific person:

*'His name was Doctor A, and he really understood me, and it felt like, you know, he was kind of on a journey with me. So, I was ... I felt like I was getting somewhere with him when new symptoms came up.'* (P13)

CFS/ME and fibromyalgia were both experienced as complex, long-term conditions often existing with comorbidities; informational continuity, or the use of past events and personal circumstances to make current care appropriate for the individual, was beneficial for understanding this complexity. Remote consulting was generally felt to be less conducive to addressing a wider holistic picture, better placed for distinct issues or limited to ‘*one problem*’, conceptually aligning with Foucault’s biomedically focused ‘medical gaze’. Two patients (P4 and P10) had used online consultation platforms to request ‘*sick notes*’ but they were not reviewed more generally:

*'You know just to check in, you know, especially my previous GP practice had been sort of basically giving me sick notes for almost 12 months and not once I get a call to just review how I was getting on*.' (P10)

Face-to-face consulting was generally felt to be more appropriate when considering the interconnected nature of these conditions and previous illness. Some participants reported having longer appointments, but physicality and non-verbal cues were again considered important and prompted clinicians to ask about the wider picture. However, a face-to-face consultation that did not address this complexity was still not regarded positively:

*'But I do think, when it’s a long-term condition, and especially a condition that’s as complex as fibro, with so many different aspects to it, including a mental health aspect, face to face is, is better, if the face-to-face person is actually up to the job.'* (P2)

Again, recognition of this complexity and integration with any consultation modality was dependent on the clinician and their knowledge of CFS/ME or fibromyalgia. There was a sense many participants had experiences with clinicians who did not seem to have a good understanding of these conditions, adding to the frustration felt with clear ‘power-knowledge’ imbalance. Having a relationship and continuity with a familiar, knowledgeable clinician therefore went some way to mitigating some of the concerns around complexity.

## Discussion

### Summary

CFS/ME and fibromyalgia were experienced as chronic, complex conditions that benefitted from a biographical approach. While face-to-face consulting was seen as a gold standard by many, it was clear to see how previous negative encounters from these interactions affected the perception and outcomes of subsequent consultations conducted remotely. Considering remote consultations with this Foucauldian lens, particularly with the additional context of much broader stigmas surrounding these conditions, underpinned the understanding of experiences. Our participants felt they needed to ‘*prove*’ symptoms within a medical system that did not bestow legitimacy on these diagnoses, highlighting a clear power imbalance and likely further contributing to negative experiences in remote spaces.

More positive experiences came from those who felt care was connected, either with a consistent clinician or by feeling that their illness experience was understood. This was particularly relevant for those with comorbid conditions viewed as related to their CFS/ME and fibromyalgia; a ‘*one consultation, one problem*’ approach was felt to insufficiently capture this complexity. Telephone and online consulting were deemed less suitable for capturing this wider narrative and integrating it into a collaborative outcome. Despite this, a positive relationship with a clinician was possible across all consulting modalities suggesting clinician approach could outweigh some of the limitations of remote consulting. The more informal use of remote consulting, particularly telephone, to ‘*check-in*’ was seen positively. When participants felt listened to and that their interconnected understanding of their illness experience was considered, consultations were seen as of value. Feeling heard, valued, and believed was at the crux of every encounter whether conducted remotely or face to face.

The exploration of experiences for those living with CFS/ME and fibromyalgia provides context for transferable findings; communication, building relationships, and managing complexity in remote spaces are applicable across patient groups. While direct comparison may be made to similar conditions, such as Long COVID, findings may also be relevant for those with non-visible disabilities (particularly of ‘*being believed*’) or other long-term conditions.

HL, an academic clinician, undertook all interviews herself and therefore steps were taken to reduce any impact this would have. She had previously undertaken an exploration of doctor–patient relationships in CFS/ME using a historical lens, so understood some of the challenges faced by many people with these conditions in healthcare spaces. HL worked closely with the experienced wider research team at multiple stages. The multiple PPI workshops were integral in ensuring lived experience and understanding influenced the study throughout its development and data analysis.

There is no doubt remote consulting is likely to remain a part of primary care systems going forward. Our participants presented varied views regarding remote consulting for those living with CFS/ME and fibromyalgia. While remote consultations could facilitate access when unwell, this had to be carefully balanced with feeling *‘seen*’ in their illness experience. There was, however, suggestion that a positive therapeutic relationship with a clinician could be maintained and experienced, outweighing some of the limitations of consulting remotely.

For those living with CFS/ME and fibromyalgia, believing their experiences and building relationships are at the heart of good care; listening and working collaboratively are possible and should be considered within any consultation modality.

### Strengths and limitations

This study provides valuable analysis of remote consulting, especially considering its evolving role in the future of general practice. It shares the voices of people who have had difficulties within health care in the hope of improving their experiences going forward. The recognition of the importance, and subsequent inclusion of, lived experience throughout the study reinforced those voices and enabled the inclusion of highly symptomatic participants with flexibility in the interviewing processes.

While the sample size was small, interview data were rich and allowed for in-depth analysis. Recruitment was conducted through general practices to identify those who were accessing services and to minimise the risk of digital health inequalities, but many people with CFS/ME and fibromyalgia (as detailed in this study) manage independently without accessing health care regularly. Future research might consider alternative recruitment strategies, including the use of third-sector organisations. Online options to respond to participant invitations could also mitigate any issues around posting replies to the research team. The participants represented a range of ages, socioeconomic backgrounds, and sexes but only one participant was of Black British Caribbean ethnicity. Previous work has considered Black and Minority Ethnic (BME) populations may be less likely to receive a diagnosis and more likely to choose to manage their symptoms outside of primary care.^21,22^ Further exploration with this patient cohort would be of benefit in the future.

### Comparison with existing literature

Some of the findings from this study are not exclusive to this patient population. Remote consulting has been found to enhance the convenience of access for many patients albeit not universal.^23^ This work also further adds to the literature on video consulting, currently used infrequently in primary care, which might enhance patient experiences through non-verbal communication.^24,25^ A recent systematic review highlighted minimal research into the relationship between remote consulting and continuity of care, which is another area that the present study adds to.^26^ Given the suggestion within this work that the clinician approach could outweigh some aspects of the modality of consultation for these complex conditions, this work also adds to the understanding of the interaction (including empathy) between clinician and patient in the remote space.^5,27^

There is extensive literature exploring the experiences of people living with CFS/ME and fibromyalgia detailing the substantial impact that these diagnoses have.^28^ Qualitative studies have sought to understand more about the relationships formed between doctors and patients with similar themes identified throughout this work: needing validity and recognition for their illness and often associated with continuity of care.^29,30^

### Implications for practice

Many participants highlighted practical ways to approach the complex and fluctuating nature of their conditions. These included messages that would pop up on booking an appointment such as a preferred clinician or appointment type. Flexibility was valued, although recognised as difficult given the current pressures on primary care.

While physicality was highlighted as important, this continued to be intertwined with needing to be ‘*seen to be believed*’. This work recognises the difficulties surrounding CFS/ME and fibromyalgia, particularly the lack of diagnostic tests or treatment. The findings reiterate and strengthen the current clinical guidance surrounding person-centred care, developing relationships, and working collaboratively, and suggest it may be achieved by clinicians across consultation modalities.

## Data

The dataset relied on in this article is available from the corresponding author on reasonable request.
